# Assessing the in Planta Efficacy of Oxathiapiprolin as a Potential Treatment for Kauri Dieback Disease

**DOI:** 10.1002/mbo3.70066

**Published:** 2025-10-08

**Authors:** Hannah F. Robinson, Jade T. T. Palmer, Monica L. Gerth

**Affiliations:** ^1^ School of Biological Sciences Victoria University of Wellington Wellington New Zealand

**Keywords:** disease management, in planta efficacy, protective treatment, Zorvec

## Abstract

*Phytophthora agathidicida*, a plant pathogenic oomycete, causes fatal dieback disease in New Zealand kauri trees (*Agathis australis*). Currently, few treatments exist to prevent or cure this infection. Previous research has demonstrated the potent in vitro inhibition of multiple lifecycle stages of *P. agathidicida* by the oomycide oxathiapiprolin. In this study, we have evaluated the efficacy of oxathiapiprolin in planta as either a protective or curative treatment. Kauri seedlings (1–2 years old) were treated with 10 or 50 mg of oxathiapiprolin, in the form of Zorvec Enicade, per seedling as a soil drench either before (7 days) or after (15 days) inoculation with *P. agathidicida* NZFS 3770 to test for protective and curative activities, respectively. Results showed that oxathiapiprolin treatments successfully protected the kauri seedlings from disease, with the higher dose (50 mg) demonstrating greater efficacy. However, the treatments did not cure kauri seedlings already infected with *P. agathidicida*, likely because the infection was already well‐established by the time of treatment. This study demonstrates that, while oxathiapiprolin shows protective effects against *P. agathidicida* infection in kauri seedlings, its lack of curative properties significantly limits its potential as a practical tool for managing kauri dieback disease.

## Graphical abstract

We tested the oomycide oxathiapiprolin as a protective and curative agent against kauri dieback disease (caused by *Phytophthora agathidicida*) in *Agathis australis* seedlings. Protective application of oxathiapiprolin (10 or 50 mg per seedling) effectively prevented disease, but it did not cure existing infections. While oxathiapiprolin has shown promising in vitro activity against *P. agathidicida*, this is the first study to evaluate its efficacy in kauri seedlings.

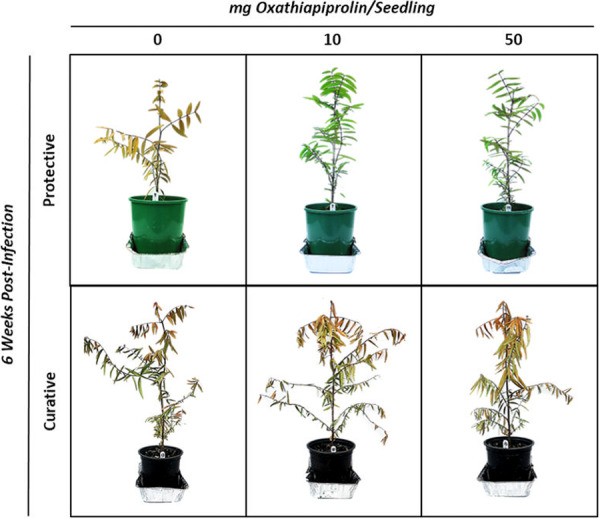

## 1 Introduction

*Phytophthora* species are plant–pathogenic oomycetes that cause significant damage to crops and natural ecosystems worldwide. Their complex, multispore lifecycle and the absence of common targets of antifungal treatments make them challenging to control chemically (Judelson and Blanco 2005; Giachero et al. 2022). Oxathiapiprolin, marketed as DuPont Zorvec, is the first member of a novel class of oomycides (piperidinyl thiazole isoxazolines) and exhibits high activity against *Phytophthora* species, providing both protective and curative effects (Ji et al. 2014; Matheron and Porchas 2014, 2015; Ji and Csinos 2015; Miao et al. 2016; Pasteris et al. 2016; Qu et al. 2016; Bittner and Mila 2017; Cohen et al. 2018, 2019; Hao et al. 2019; Cohen 2020; Cohen and Rubin 2020). Therefore, it has potential as a treatment for currently uncontrolled or difficult‐to‐manage *Phytophthora* diseases.

In New Zealand (NZ), *Phytophthora agathidicida* is the causative agent of dieback disease in endemic kauri (*Agathis australis*) (Bradshaw et al. 2020). Kauri are large, long‐lived conifer trees of ecological and cultural significance (Mainwaring et al. 2023). Kauri dieback is a lethal root rot disease characterized by the hyperproduction of resin around the tree collar and lower trunk, leaf yellowing, and canopy thinning, typically resulting in death within 10 years (Bellgard et al. 2016; Bradshaw et al. 2020). The now widespread nature of dieback disease within the natural range of kauri in the northern North Island threatens the health of kauri forests, making control of the epidemic imperative (Bradshaw et al. 2020).

Current strategies for managing kauri dieback include containment measures and chemical controls (Bradshaw et al. 2020). Containment efforts involve installing footwear washing stations at track entrances, upgrading or closing tracks in vulnerable areas, and implementing restrictions on earthworks, stock grazing, and animal release in kauri lands (Bellgard et al. 2016; Bradshaw et al. 2020; Kiro 2023). Chemical control trials have shown that phosphite injections into the trunks of infected kauri trees have a curative effect (Horner and Hough 2013; Horner et al. 2015; Horner and Arnet 2020). However, phytotoxicity at higher phosphite concentrations and the potential for resistance warrant research into alternative chemical controls with greater potency and tolerability (Dobrowolski et al. 2008; Horner et al. 2015; Horner and Arnet 2020).

Oxathiapiprolin is a promising candidate for the chemical control of *P. agathidicida*. It can be applied as a soil drench to manage soilborne diseases, such as kauri dieback, and its acropetal and xylem systemic movement protects growing leaves and new foliage from infection (Pasteris 2019). Oxathiapiprolin targets the oomycete oxysterol binding protein, which plays roles in sterol transport, lipid metabolism, and lipid membrane composition, impacting signaling and vesicle transport (Weber‐Boyvat et al. 2013; Pasteris 2016, 2019). Thus, oxathiapiprolin inhibits multiple stages of the pathogen's lifecycle and has specific antioomycete activity, minimizing off‐target effects. Specificity is especially important for chemical controls in kauri habitats, and studies have confirmed that oxathiapiprolin has favorable environmental and toxicological profiles (Pasteris 2016, 2019).

In previous work, our lab group has reported the potent in vitro inhibition of the three major lifecycle stages of *P. agathidicida* (mycelial growth, zoospore germination, and oospore germination) by oxathiapiprolin at sub‐microgram per milliliter concentrations (Lacey et al. 2021). The inhibition of zoospore and oospore germination suggests a potential protective benefit of oxathiapiprolin against *P. agathidicida* in kauri, while the inhibition of mycelial growth indicates possible curative properties. Therefore, in this study, we conducted plant trials with kauri seedlings to determine the optimal dosage of oxathiapiprolin and assess its protective and/or curative effects following inoculation with *P. agathidicida*. This paper summarizes the in planta activity of oxathiapiprolin when applied as a soil drench.

## 2 Materials and Methods

### 2.1 *P. agathidicida* Isolate and Culture Conditions

*P. agathidicida* NZFS 3770 (ICMP 17027 holotype) was provided from Scion's culture collection (Rotorua, NZ) but was originally isolated in NZ from Great Barrier Island (Guo et al. 2020). Upon receipt, it was initially cultured on oomycete‐selective pimaricin, ampicillin, rifampicin, pentachloronitrobenzene agar plates composed of cornmeal agar (17 g/L; BD BBL, Sparks, MD, USA) supplemented with pimaricin (0.001% w/v; Sigma‐Aldrich, St. Louis, MO, USA), ampicillin (250 µg/mL; GoldBio, St. Louis, MO, USA), rifampicin (10 µg/mL; Sigma‐Aldrich), and pentachloronitrobenzene (100 µg/mL; Sigma‐Aldrich). Subsequent cultures were passaged by transferring an agar plug with a cork borer from the leading edge of mycelial growth onto 20% v/v clarified V8 (cV8) agar plates. Clarified V8 agar (1 L) was prepared by mixing 200 mL V8 Original Vegetable Juice with 200 mL distilled, deionized water (ddH_2_O) and 2 g CaCO_3_ for 30 min and then clarifying via centrifugation at 7000*g* for 10 min. Bacteriological agar (15 g; New Zealand Seaweeds, Opotiki, NZ) and 600 mL ddH_2_O were added to the supernatant, which was thereafter sterilized by autoclaving. All cultures were grown in the dark at 22°C.

### 2.2 Preparation and Maintenance of Kauri Seedlings

For the protective plant trial, kauri (*A. australis*) seedlings (1–2 years old, 30–70 cm tall) obtained from Ōtari‐Wilton's Bush (Wellington, NZ) after germination and subsequently grown in a glasshouse were repotted in horticultural polypropylene pots (2 L/15 cm diameter) using Tui Essentials All Purpose Potting Mix. The repotted seedlings were left in the glasshouse for ≥ 1 week to ensure they were undamaged from the repotting. For the curative plant trial, kauri seedlings (approximately 2 years old, 50–80 cm tall, 1.5 L pots) purchased from Naturally Native (Tauranga, NZ) were left in the glasshouse for ≥ 1 week to acclimatize. Before beginning the plant trials, all seedlings were transferred to a plant containment facility, in which they were allowed to equilibrate for ≥ 1 week. The plant lab was maintained at 18°C–22°C (35%–75% relative humidity). The seedlings were placed in individual aluminum trays and watered every 2–3 days until damp but not waterlogged. They were grown under fluorescent tube lights (Growshop 4 ft 4 × 54 W T5 Fluorescent Tube Fitting c/w 6500k) on a 12 h on: 12 h off cycle.

### 2.3 Treatment Preparation and Application

Zorvec Enicade (ZE), containing 100 g oxathiapiprolin/L as an oil dispersion, was provided by Corteva Agriscience (New Plymouth, NZ) and stored at room temperature. Three seedling replicates were prepared for each treatment and control type. Treatment types included seedlings inoculated with *P. agathidicida* (PA) and treated with 10 or 50 mg oxathiapiprolin/seedling (+PA, +ZE), and control types included seedlings that were uninoculated and untreated (−PA, −ZE), uninoculated but treated with 50 mg oxathiapiprolin/seedling (−PA, +ZE), and inoculated but untreated (+PA, −ZE). Seedlings were treated with 10 or 50 mg oxathiapiprolin/seedling by diluting ZE in sterile ddH_2_O to final concentrations of 0.2 or 1 mg oxathiapiprolin/mL (total volume = 50 mL), respectively, and pouring each 50 mL treatment in the root zone of the corresponding seedling, as in Horner and Hough (2013). Each pot was placed in a plastic bag that was tied around the bottom of the stem to reduce evaporation, and the seedlings were not watered for 1 week to allow time for the uptake of each treatment. Sterile ddH_2_O (50 mL) was applied to the untreated control seedlings (±PA, −ZE). For the protective plant trial, the seedlings were treated 1 week before the first inoculation, and for the curative plant trial, the seedlings were treated 1 day after the third (and final) inoculation (i.e., 15 days after the first inoculation).

### 2.4 Preparation of Inocula and Kauri Seedling Inoculation

The inocula were prepared as follows: agar plugs (4 mm^2^) from the leading edge of *P. agathidicida* NZFS 3770 mycelial growth on 20% v/v cV8 agar were incubated in 10 mL of 2% v/v cV8 broth at 22°C in the dark for 2 days. For the uninoculated control seedlings (−PA, ±ZE), blank 20% v/v cV8 agar plugs (i.e., no *P. agathidicida* present) were prepared in the same manner. As in Byers et al. (2021), kauri seedlings were inoculated with five mycelial mats (or blank cV8 agar plugs for the uninoculated control seedlings) as follows: 8 mm diameter wooden dowels were used to create five evenly spaced, 5–10 cm deep holes in the soil around the seedling within the root zone. The 10 mL mycelial cultures (or blanks) were poured into the holes and then covered with soil, and the seedlings were watered daily until total saturation for 1 week to promote zoospore motility and stimulate infection. The seedlings were reinoculated twice with three mycelial mats (or blank cV8 agar plugs) after 1 and 2 weeks to ensure infection and then saturated with water overnight. The seedlings were monitored weekly by taking pictures, measuring seedling height, and recording observations.

### 2.5 Assessment of Disease Symptoms

Six weeks postinoculation, the seedlings were randomized, and five blinded assessors independently scored disease symptoms. The assessment criteria, based on documented disease progression, included initial wilting of foliage; progressive color changes from green to yellow (chlorosis), then brown and orange; wilting and drying of all leaves; and reduction of new healthy growth. Disease severity was scored on a scale where higher values indicated more severe symptoms.

*Wilting:* Assessment of the overall severity of leaf wilting for each seedling.

**Table:** 

Rating	Description
0	No wilting
1	Mild wilt symptoms
2	Moderate wilt symptoms
3	Severe wilt symptoms

*Color:* Assessment of the overall extent of browning/yellowing in the leaves of each seedling.

**Table:** 

Rating	Description
0	No browning/yellowing (i.e., all leaves green)
1	Mild browning/yellowing
2	Moderate browning/yellowing
3	Severe browning/yellowing

*Dryness:* Estimation of the percent of leaves (or total leaf material) that have dried out in each seedling.

**Table:** 

Rating	Description (%)
0	0
1	> 0–≤ 10
2	> 10–≤ 50
3	> 50–< 100
4	100

*New Foliage:* Observation of the presence and health of new growth.

**Table:** 

Rating	Description
0	New, healthy growth is present
1	No new growth is present or new growth is unhealthy

Mean severity scores were calculated for each seedling across the five blinded assessors for subsequent analysis. The results were analyzed on GraphPad Prism 8.4.3 using a two‐way factorial analysis of variance with the Tukey post hoc correction to determine significant differences (*p* ≤ 0.05).

### 2.6 Interrater Reliability

To confirm that the assessors consistently applied the criteria for wilting (scored 0–3), color (0–3), dryness (0–4), and new foliage (0–1), interrater reliability was evaluated using Krippendorff's alpha (*α*). A value of 0.8 or higher was considered indicative of an acceptable level of agreement, while *α* ≥ 0.667 represented the minimum threshold at which tentative conclusions remained acceptable (Krippendorff 2004). These calculations were made using R version 4.1.2 for Windows and the kripp.alpha function (irr: Various Coefficients of Interrater Reliability and Agreement. R package version 0.84.1).

## 3 Results

### 3.1 Efficacy of Oxathiapiprolin as a Protective Treatment Applied to Kauri Seedlings via Soil Drench

**Figure 1:**
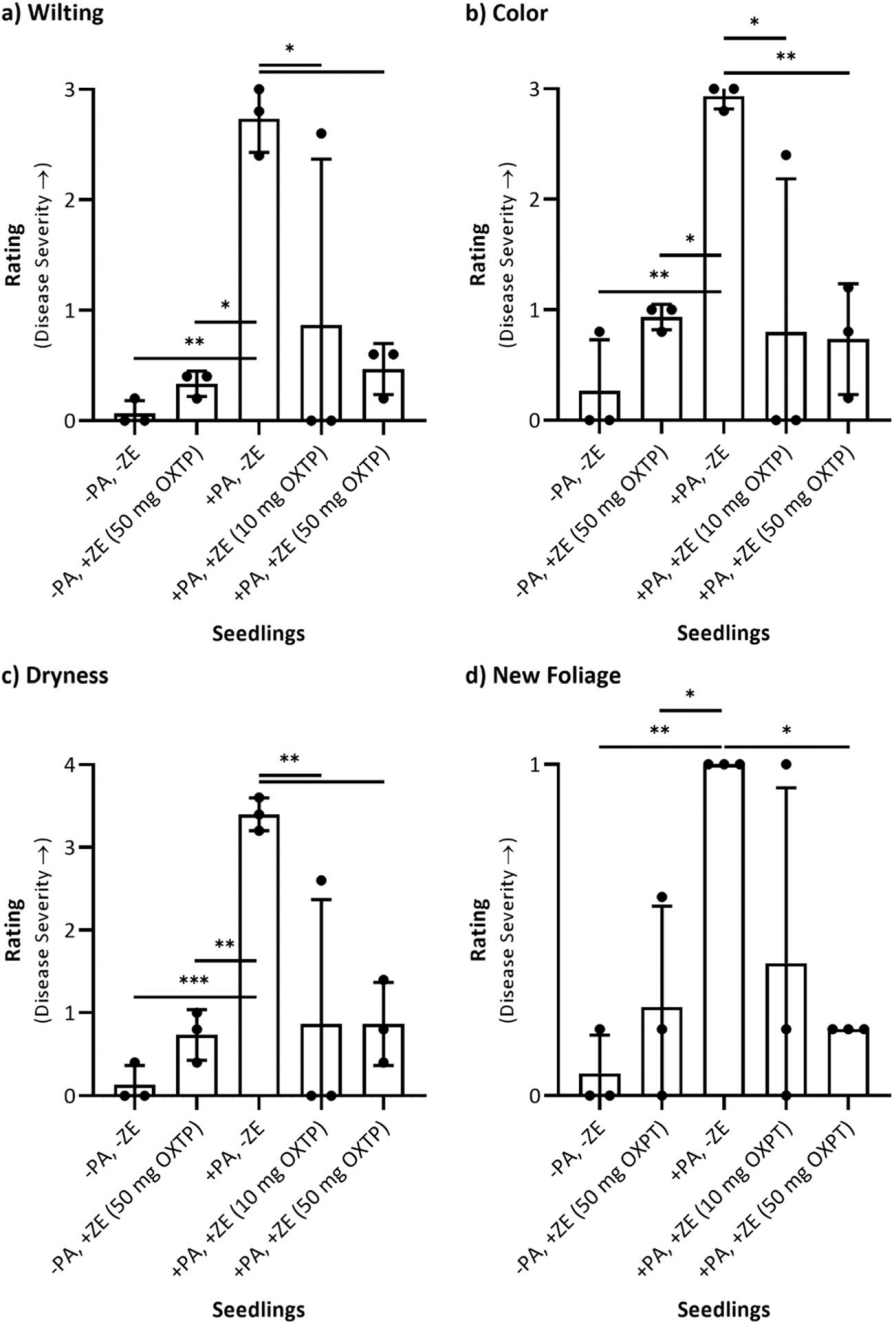
Results of the five blinded, independent ratings of (a) wilting, (b) color, (c) dryness, and (d) new foliage in the kauri seedlings treated protectively with Zorvec Enicade (ZE) (10 or 50 mg oxathiapiprolin/seedling). The bars represent the mean (*n* = 3) ± standard deviation. To determine if the means from the data sets were significantly different from each other, a two‐way factorial analysis of variance with the Tukey post hoc correction was conducted, and the difference was considered to be significant when the *p* value was ≤ 0.05 (*p ≤ 0.05; **p ≤ 0.01; ***p ≤ 0.001). Unless annotated, the difference was not considered to be significant. OXTP = oxathiapiprolin; ± PA = inoculated or not inoculated with *Phytophthora agathidicida* NZFS 3770; ± ZE = treated or not treated with ZE.

**Figure A1:**
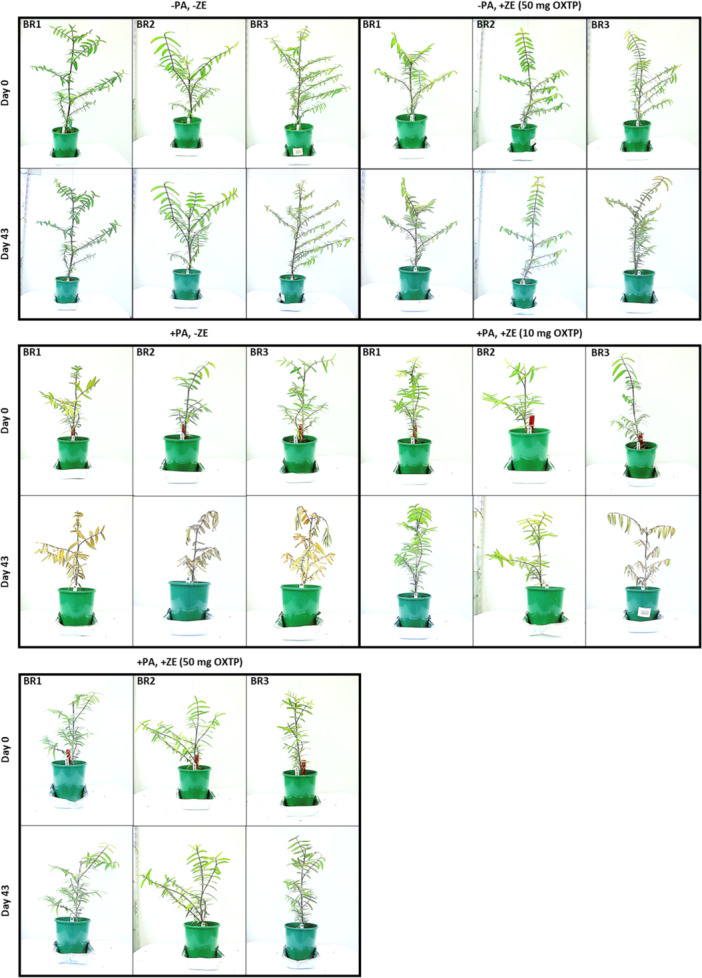
Kauri seedlings on days 0 and 43 treated protectively with Zorvec Enicade (ZE) (10 or 50 mg oxathiapiprolin/seedling). Brightness, contrast, and saturation were adjusted in the pictures for better visualization of effect. BR = biological replicate; OXTP = oxathiapiprolin; ± PA = inoculated or not inoculated with *Phytophthora agathidicida* NZFS 3770; ± ZE = treated or not treated with ZE.

Kauri seedling health was assessed 6 weeks after the initial inoculation with *P. agathidicida* NZFS 3770, following protective treatment of 10 or 50 mg oxathiapiprolin applied as a soil drench (Figures 1 and A1). Interrater reliability was acceptable for wilting (*α* = 0.748), color (*α* = 0.891), and dryness (*α* = 0.806). While the interrater agreement for new foliage (*α* = 0.580) was lower, primarily due to one outlier rater and a single ambiguous seedling, these results are shown for completeness.

No significant differences were observed between uninoculated/untreated seedlings (−PA, −ZE) and uninoculated/treated seedlings (−PA, +ZE) across all rating categories (Figure 1). This indicates that the soil drench application of ZE (50 mg oxathiapiprolin per seedling) did not affect seedling health, and that the treatment was not phytotoxic.

Inoculated/untreated seedlings (+PA, −ZE) exhibited significantly higher disease severity scores (*p* < 0.05) across all rating categories (wilting, color, dryness, and new foliage) compared with uninoculated seedlings (−PA). This confirmed the pathogenicity of *P. agathidicida* in causing dieback symptoms in untreated kauri seedlings.

Inoculated/treated seedlings (+PA, +ZE) demonstrated significantly lower disease severity ratings (*p* < 0.05) compared with inoculated/untreated seedlings (+PA, −ZE) in the wilting (Figure 1A), color (Figure 1B), and dryness categories (Figure 1C) at both treatment dosages tested. Additionally, seedlings treated with 50 mg oxathiapiprolin (+PA, +ZE [50 mg OXTP]) exhibited increased emergence of new, healthy foliage, leading to a significantly lower disease severity score for new foliage (*p* < 0.05; Figure 1D). These findings indicate that ZE, applied as a soil drench at both tested dosages, effectively protected kauri seedlings from *P. agathidicida* infection, with the higher dose providing greater protection.

### 3.2 Efficacy of Oxathiapiprolin as a Curative Treatment Applied to Kauri Seedlings via Soil Drench

**Figure 2:**
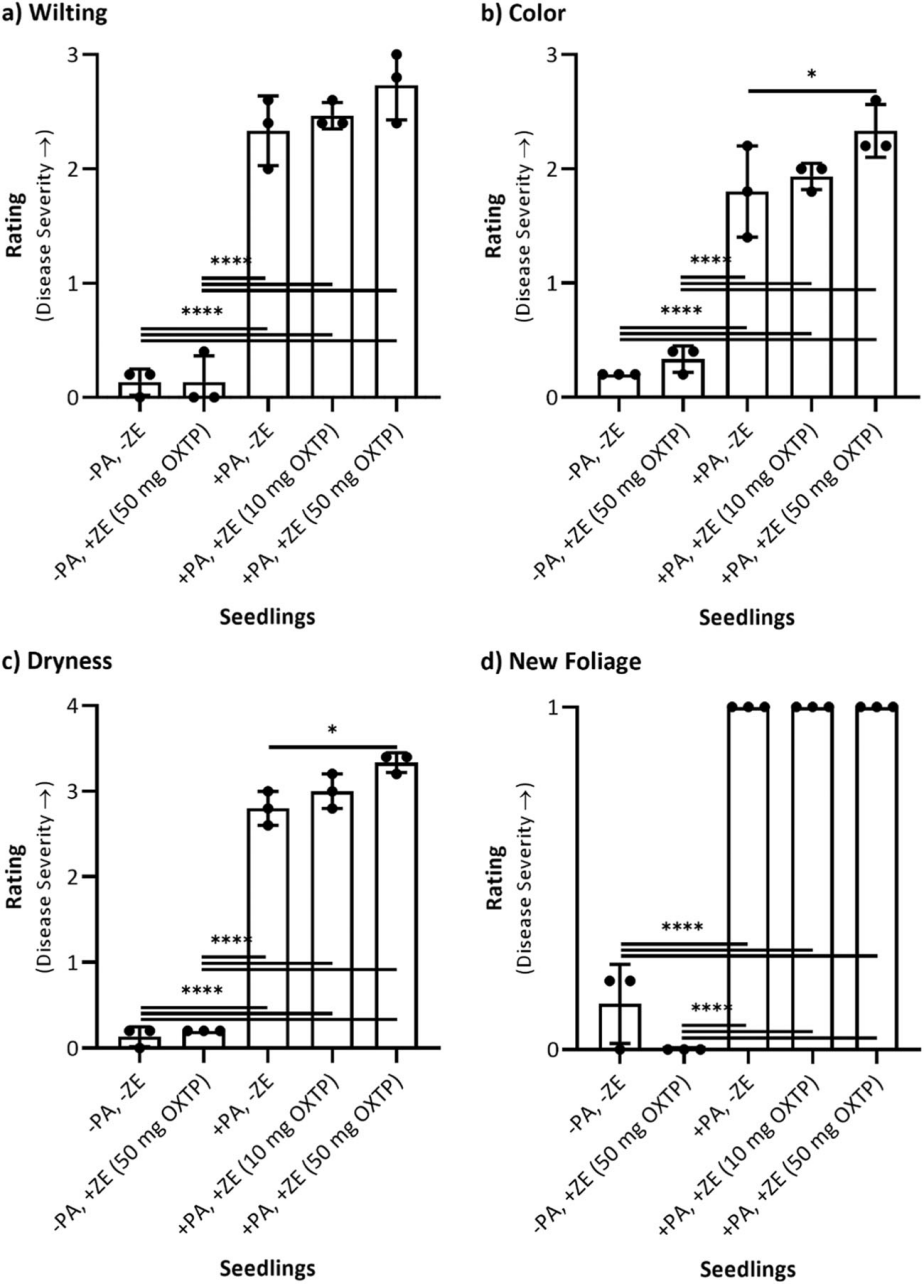
Results of the five blinded, independent ratings of (a) wilting, (b) color, (c) dryness, and (d) new foliage in the kauri seedlings treated curatively with Zorvec Enicade (ZE) (10 or 50 mg oxathiapiprolin/seedling). The bars represent the mean (*n* = 3) ± standard deviation. To determine if the means from the data sets were significantly different from each other, a two‐way factorial analysis of variance with the Tukey post hoc correction was conducted, and the difference was considered to be significant when the *p* value was ≤ 0.05 (*p ≤ 0.05; **p ≤ 0.01; ***p ≤ 0.001; ****p ≤ 0.0001). Unless annotated, the difference was not considered to be significant. OXTP = oxathiapiprolin; ± PA = inoculated or not inoculated with *Phytophthora agathidicida* NZFS 3770; ± ZE = treated or not treated with ZE.

**Figure A2:**
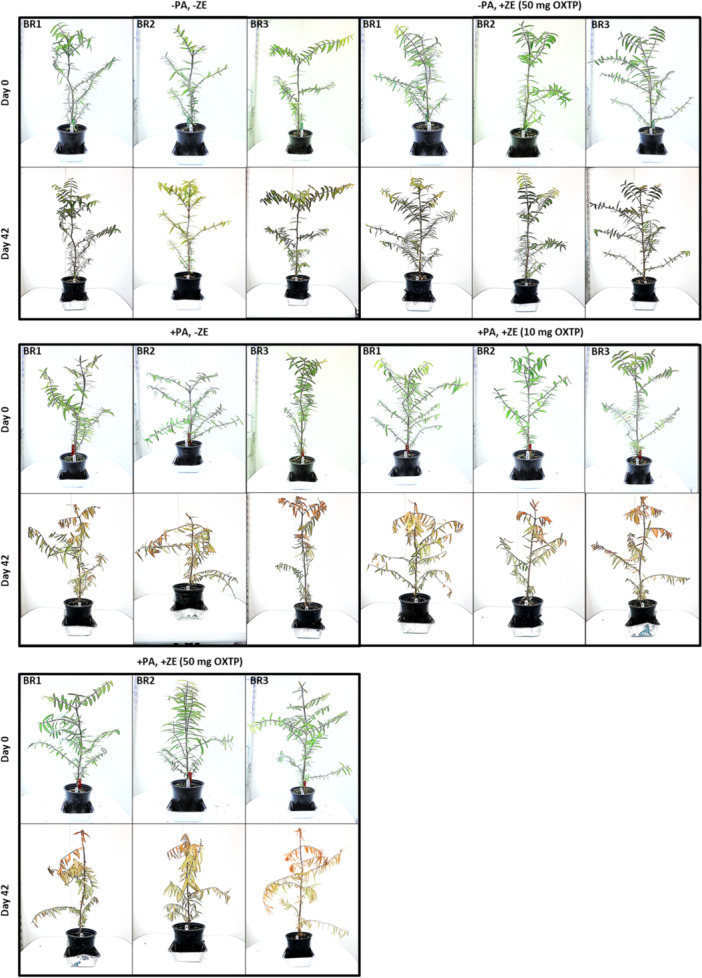
Kauri seedlings on days 0 and 42 treated curatively with Zorvec Enicade (ZE) (10 or 50 mg oxathiapiprolin/seedling). Brightness, contrast, and saturation adjusted in the pictures for better visualization of effect. BR = biological replicate; OXTP = oxathiapiprolin; ± PA = inoculated or not inoculated with *Phytophthora agathidicida* NZFS 3770; ± ZE = treated or not treated with ZE.

In a separate plant trial, the curative efficacy of oxathiapiprolin, applied as a soil drench (10 or 50 mg per seedling), was evaluated on kauri seedlings already inoculated with *P. agathidicida*. At the time of treatment, six of the nine inoculated seedlings exhibited initial wilting of new foliage, consistent with early disease symptoms. Disease progression was assessed 6 weeks postinoculation (Figures 2 and A2). Interrater reliability among five blinded assessors was satisfactory for all criteria: wilting (*α* = 0.798), color (*α* = 0.704), dryness (*α* = 0.766), and new foliage (*α* = 0.886).

No significant differences were found in any symptom category between uninoculated/untreated seedlings (−PA, −ZE) and uninoculated/treated seedlings (−PA, +ZE), confirming that soil drench application of ZE was not phytotoxic (Figure 2). Disease severity ratings for wilting, color, dryness, and new foliage were significantly higher (*p* < 0.05) for all seedlings inoculated with *P. agathidicida* (+PA) when compared with uninoculated controls, regardless of treatment status (Figure 2). These findings are consistent with those observed in the preceding protective trial.

Unexpectedly, seedlings treated with 50 mg oxathiapiprolin exhibited more severe disease symptoms. Among inoculated seedlings, those treated with 50 mg oxathiapiprolin (+PA, +ZE at 50 mg OXTP) showed significantly (*p* < 0.05) greater severity in color (increased browning/yellowing; Figure 2B) and dryness (more dried leaves; Figure 2C) than untreated inoculated seedlings (+PA, −ZE). Overall, soil drench application of ZE at both 10 and 50 mg oxathiapiprolin per seedling did not demonstrate curative effects on kauri seedlings already infected with *P. agathidicida*.

## 4 Discussion

The experimental design of these plant trials (e.g., inoculation and treatment application methods, treatment amount) was informed by previous method development trials, as well as plant trials with oxathiapiprolin or kauri in the literature (Horner and Hough 2013; Hao et al. 2019; Byers et al. 2021; Davison and Hardy 2023). Consistent with Byers et al. (2021), we employed a sustained infection protocol, inoculating kauri seedlings weekly for 3 weeks by pouring mycelial mats into the root zone and saturating the soil to facilitate zoospore motility. The observed severe symptoms or mortality in all diseased seedlings by 6 weeks after the initial inoculation demonstrated the significant virulence of *P. agathidicida*, thus establishing this time point as the appropriate end for the trials. This aligns with Byers et al. (2021), who also reported dieback symptoms (chlorosis, wilting, leaf litter, necrosis, and mortality) in 18‐month‐old kauri seedlings, 6 weeks after infection by *P. agathidicida* NZFS 3770.

The selection of a soil drench application for oxathiapiprolin was based on the understanding that *P. agathidicida* is a root rot pathogen and oxathiapiprolin exhibits systemic, acropetal movement via the xylem, but lacks phloem translocation (Pasteris 2016, 2019). Horner and Hough (2013) also found that applying phosphite to kauri seedlings as a soil drench improved root and foliar health and tree survival for seedlings soil‐inoculated with *P. agathidicida*; in contrast, foliar sprays were ineffective. Although soil drenching was the chosen method for these trials, future investigations into alternative application methods may be warranted.

**Figure A3:**
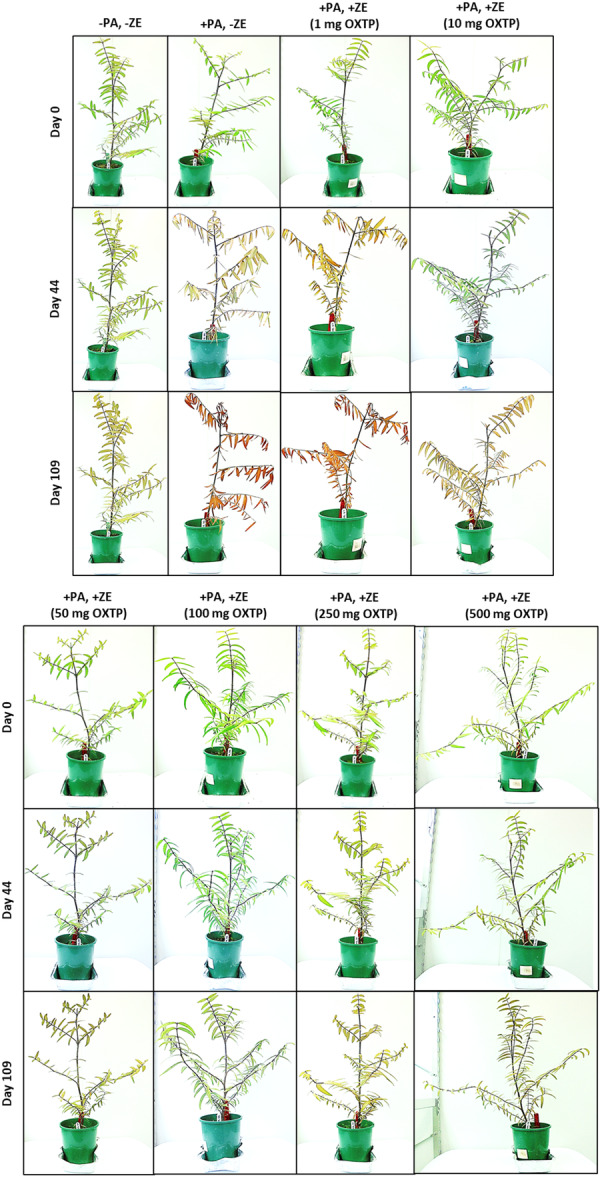
Kauri seedlings on days 0, 44, and 109 treated protectively with Zorvec Enicade (ZE) (1, 10, 50, 100, 250, or 500 mg oxathiapiprolin/seedling). Brightness, contrast, and saturation adjusted in the pictures for better visualization of effect. OXTP = oxathiapiprolin; ± PA = inoculated or not inoculated with *Phytophthora agathidicida* NZFS 3770; ± ZE = treated or not treated with ZE.

In a method development experiment, we initially tested a range of amounts of oxathiapiprolin per kauri seedling (1, 10, 50, 100, 250, and 500 mg) applied as a soil drench to determine the optimal dosage and found that 10 mg was the lowest amount to demonstrate a protective effect against infection by *P. agathidicida* NZFS 3770 (Figure A3). Therefore, we selected this amount, as well as the next highest amount of 50 mg, for plant trials moving forward. Although oxathiapiprolin has potent in vitro activity against multiple lifecycle stages of *P. agathidicida* at sub‐microgram per milliliter concentrations (Lacey et al. 2021), the necessity to upscale and apply milligram quantities of oxathiapiprolin to the seedlings was in agreement with expectations based on the literature: in these studies, microgram‐to‐milligram quantities of oxathiapiprolin were applied to field or vegetable crop plants, such as tomato, tobacco, and pepper plants, to prevent and/or cure infection by *Phytophthora* species (Ji et al. 2014; Matheron and Porchas 2015; Miao et al. 2016; Bittner and Mila 2017; Cohen et al. 2018; Cohen 2020; Cohen and Rubin 2020). More closely aligned with our work, Hao et al. (2019) performed greenhouse studies of 6–9‐month‐old citrus seedlings (‘Madam Vinous’ sweet orange) inoculated with root rot pathogens *Phytophthora nicotianae* or *Phytophthora citrophthora* and treated 1 week later with a soil drench of 8 mg oxathiapiprolin/seedling, and they found that the incidence of root rot and the pathogen populations in the soil were reduced to zero or near zero.

Our findings demonstrate that oxathiapiprolin, at 10 mg and more effectively at 50 mg per seedling, applied as a soil drench, provides significant protective effects against dieback disease in kauri seedlings challenged with *P. agathidicida* NZFS 3770. However, the treatment was not curative. Both protective and curative effects of oxathiapiprolin against oomycete infections in plants have been reported (Ji et al. 2014; Matheron and Porchas 2014, 2015; Ji and Csinos 2015; Miao et al. 2016; Bittner and Mila 2017; Cohen et al. 2018, 2019; Salas et al. 2019; Hao et al. 2019; Cohen 2020; Cohen and Rubin 2020). However, the extent of these effects can vary. For instance, Miao et al. (2016) applied oxathiapiprolin to pepper plants preinoculation (protective) or postinoculation (curative) with *Phytophthora capsici*. These treatments demonstrated increased control of *P. capsici* infection, whether applied before or after inoculation, but the protective activity was reported to be greater than the curative activity. Similarly, Cohen et al. (2018) reported that curative application of oxathiapiprolin was considerably less effective than protective application in controlling *Phytophthora infestans* in tomato plants. Furthermore, commercial recommendations for Zorvec emphasize its preventative use (Luijks 2017). In our curative trial, the presence of initial wilting in two‐thirds of the inoculated seedlings at the time of treatment suggests that the infection may have been too advanced for effective cure (aboveground symptoms often manifest long after substantial root damage and pathogen biomass accumulation, Ivic 2010). While our study relied on visual assessment of symptoms, future work could incorporate molecular (e.g., quantitative polymerase chain reaction) or microscopic techniques to directly quantify pathogen load within the plant tissue, providing a more precise measure of infection level and treatment efficacy, particularly for curative applications.

## 5 Conclusions

Our results show that oxathiapiprolin (10 or 50 mg/seedling) applied as a soil drench effectively protects kauri seedlings from *P. agathidicida* NZFS 3770 infection, but it is not curative once dieback symptoms are present. Oxathiapiprolin could have limited usefulness as a protective treatment in plant nurseries and for private landowners, but its use in a forest setting is less practical, facing the same challenges as other chemical controls: the determination of the appropriate dosage and required frequency of application, avoiding phytotoxicity and off‐target effects, and the feasibility of treatment application (e.g., amount needed to cover large trees, cost, method, reaching the forest sites for repeated treatments). Before evaluating the capability of oxathiapiprolin to protect larger trees, further seedling trials will be needed to assess different application methods and *P. agathidicida* isolates, as well as the protective activity on a longer timescale with multiple reinoculations. Oxathiapiprolin could also be tested as a curative treatment when applied before the first disease symptoms. Furthermore, since oxathiapiprolin is at high risk for the development of resistance due to its single‐site mode of action, it would be prudent to evaluate the activities of next‐generation dual treatments that combine oxathiapiprolin with other oomycides (e.g., mefenoxam, famoxadone, benthiavalicarb, and mandipropamid) to combat resistance.

## Author Contributions

**Hannah Robinson:** methodology (lead), formal analysis (lead), investigation (lead), supervision (supporting), validation (lead), visualization (lead), writing – original draft (lead), writing – review and editing (lead). **Jade Palmer:** investigation (supporting), writing – review and editing (supporting). **Monica Gerth:** conceptualization (lead), methodology (supporting), funding acquisition (lead), project administration (lead), resources (lead), supervision (lead), writing – review and editing (supporting).

## Ethics Statement

The authors have nothing to report.

## Conflicts of Interest

The authors declare no conflicts of interest.

## Data Availability

All data sets from the current study, including pictures and raw and processed data, are openly available in the Zenodo repository at https://zenodo.org/, reference number 10.5281/zenodo.13831388.
